# A mechanism underlying improved dual-task performance after practice: Reviewing evidence for the memory hypothesis

**DOI:** 10.3758/s13423-024-02498-0

**Published:** 2024-03-26

**Authors:** Torsten Schubert, Sebastian Kübler, Tilo Strobach

**Affiliations:** 1 Department of Psychology, Martin-Luther University Halle-Wittenberg, Emil-Abderhalden-Str. 26-27, 06108 Halle, Saale, Germany; 2 Humboldt-Universität zu Berlin, Berlin, Germany; 3 Department of Psychology, Medical School Hamburg, Am Kaiserkai 1, 20457 Hamburg, Germany; 4 ICAN Institute for Cognitive and Affective Neuroscience, Medical School Hamburg, Am Kaiserkai 1, 20457 Hamburg, Germany

**Keywords:** Dual tasks, Dual-task performance, Dual-task practice, Task coordination, Coordination skills

## Abstract

Extensive practice can significantly reduce dual-task costs (i.e., impaired performance under dual-task conditions compared with single-task conditions) and, thus, improve dual-task performance. Among others, these practice effects are attributed to an optimization of executive function skills that are necessary for coordinating tasks that overlap in time. In detail, this optimization of dual-task coordination skills is associated with the efficient instantiation of component task information in working memory at the onset of a dual-task trial. In the present paper, we review empirical findings on three critical predictions of this memory hypothesis. These predictions concern (1) the preconditions for the acquisition and transfer of coordination skills due to practice, (2) the role of task complexity and difficulty, and (3) the impact of age-related decline in working memory capacity on dual-task optimization.

## Introduction

Performing two tasks simultaneously often comes at a cost compared with performing the same tasks in isolation (e.g., Telford, 1931; Welford, 1952). However, extensive practice can significantly reduce these dual-task costs and, thus, improve dual-task performance (e.g., Spelke et al., 1976; Van Selst et al., 1999). According to some accounts, these practice effects can be attributed to an optimization of executive functions that are necessary for coordinating the two task processing streams that overlap in time (Damos & Wickens, 1980; Hirst et al., 1980; Liepelt et al., 2011). However, the exact mechanisms underlying this optimization are hardly understood. To tackle this issue, we review findings on a hypothesis according to which practice-related optimization results from the efficient instantiation of information for more than one component task in working memory at the onset of a dual-task trial: the memory hypothesis for dual-task practice (Schubert & Strobach, 2018; Strobach et al., 2014).

As will be specified below, the memory hypothesis integrates assumptions about the functionality of the working memory system, the maintenance of the component task representations, the operation of their coordination during dual-task processing, and assumptions about ongoing practice-related changes in dual-task processing. Based on that integration, the hypothesis makes testable predictions about the specific learning conditions during practice that allow or do not allow for acquiring skills of improved task coordination. It also allows for predictions regarding the function of a task’s memory load in determining whether or not the corresponding coordination information can be processed successfully in working memory. Finally, it predicts that differences in working memory capacity of participants can result in more or less successful acquisition of the efficient instantiation of information about the task situation in working memory. These predictions were the essence of a funded research project, starting in 2008 (https://gepris.dfg.de/gepris/projekt/106904954?language=en), and stimulated a number of research studies testing and extending the implications of the memory hypothesis. The current review is aimed at summarizing the results of these empirical studies, which worked on different aspects of the memory hypothesis, to relate the results to the main predictions of the hypothesis and, as such, to provide a more coherent view of the underlying theory.

## Dual-task processing and its optimization due to practice

**Fig. 1:**
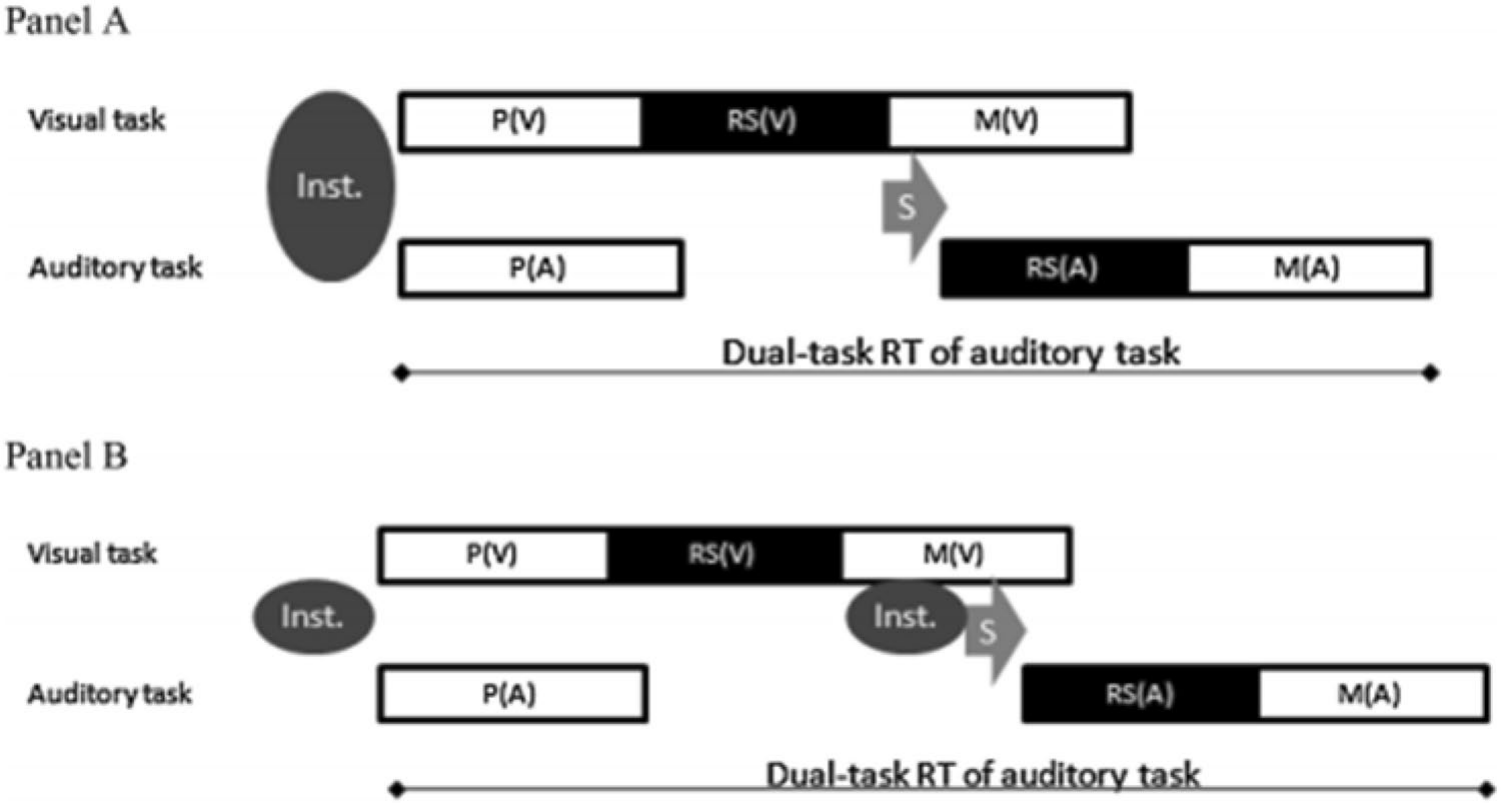
Illustration of the hypothetical time relation of dual-task processing in the visual and auditory tasks as a shorter and a longer task, respectively, when presented in a dual-task situation with a stimulus onset asynchrony of 0 ms. **Panel A:** Hypothetical time relation of dual-task processing at the end of dual-task practice, including efficient instantiation of information for two tasks and no additional instantiation processes after the completion of the response selection in the visual task—RS(V)—and before the response selection in the auditory task—RS(A)—leading to a relatively short switch between tasks and relatively fast dual-task reaction times in the auditory task. **Panel B:** Hypothetical time relation of dual-task processing without efficient instantiation of information of two tasks at the onset of a dual-task trial and additional instantiation processes after the completion of RS(V) and before RS(A), leading to relatively long switch between tasks and relatively slow dual-task reaction times in the auditory task. This relation of dual-task processing illustrates the status of task coordination at the beginning of (dual-task/single-task) practice or at the end of single-task practice. P(V) and P(A) = the perception stages; RS(V) and RS(A) = the central response-selection stages (including bottleneck characteristics); M(V) and M(A) = the motor stages; Inst. = instantiation of task information; S = switching between component tasks after the completion of RS(V) and before the start of RS(A)

We start by reviewing assumptions on potential mechanisms for the optimization of dual-task processing within the framework of the central bottleneck account. This account suggests that dual-task costs occur due to a capacity limitation at the central response selection stage (Pashler, 1994; Schubert et al., 2008; but also see Fischer & Plessow, 2015). According to this view, while the perceptual and motor processing stages operate in parallel, the response selection stage is limited in capacity and thus can only process one task at a time (Fig. 1). As a result, the processing of one of the two tasks is interrupted while the response selection stage of the other task is processed. Processing for the interrupted task can only continue after the end of the response selection for the non-interrupted tasks. The resulting waiting time for the interrupted task can explain increased reaction times (RTs) and, thus, dual-task costs.

A number of studies have shown that dual-task processing can be optimized as a result of extended practice (e.g., Liepelt et al., 2011; Ruthruff et al., 2001, 2003). In some cases, practice leads to a strong reduction, and even a complete elimination, of dual-task costs (Schumacher et al., 2001). Although the reduction of dual-task costs due to practice seems to be a reliable phenomenon, the underlying mechanisms are still a matter of debate. A plethora of different key mechanisms have been proposed, explaining a substantial amount of dual-task cost reductions, such as the elimination and bypassing of the central bottleneck stage (Maquestiaux et al., 2008, 2018; Meyer & Kieras, 1997; Ruthruff et al., 2001), the shortening of relevant processing stages (Dux et al., 2009; Ruthruff et al., 2006; Strobach et al., 2013; Thomson et al., 2015; Van Selst et al., 1999), automatization (Schneider & Shiffrin, 1977) and others; see Strobach and Schubert (2017a) for a comprehensive review of these key mechanisms.

In light of these previously proposed mechanisms, the current review paper elaborates on an additional source of reduced dual-task costs, according to which practice-related improvements in dual-task processing can be attributed to an efficient re-scheduling of different processing stages during dual-task performance. According to the memory hypothesis, optimized dual-task performance can be explained by the acquisition of skills for improved coordination of the two task processing streams at the bottleneck stage. This improvement allows for the fast instantiation of a second task (i.e., Task 2) after the processing of the first task (Task 1) has finished, which is accompanied by the need for simultaneous maintenance of two fully prepared task representations in working memory during dual-task processing. This requires sufficient separability of the task representations such that they cannot be confused in working memory (Hommel, 1998) but are maintained as processable instances. As a result, this leads to advantages in dual-task processing because of the reduction of processing time for scheduling and timing of task processes compared with situations in which the component task representations are uploaded serially, one after the other, into the working memory (Schubert & Strobach, 2018).

Several assumptions about the working memory that is involved in that process allow for specifying predictions about the conditions for the occurrence of practice-related effects on task information. In particular, task representations are held active in working memory as executable instances, which is proposed by different models assuming different representation formats (Braver & Cohen, 2000; Kimberg & Farah, 1993). The capacity for simultaneous maintenance of task representations is clearly limited, although the specific limit is certainly still a matter of debate (e.g., Cowan, 2001; Oberauer & Hein, 2012). Further, a number of active mechanisms can operate to cause proper maintenance and uploading of memory contents, such as gating and shielding (e.g., Braver & Cohen, 2000; Miyake & Shah, 1999). The integration of such assumptions about the working memory with assumptions about task processing is not a new idea and can be found in many studies on task processing and related control mechanisms. In the context of the current model of task coordination the integration allows specifying conditions under which task coordination can be improved by practice. In particular, (1) this integration predicts that the acquisition of improved task coordination skills can preferably result from situations in which participants practice two tasks simultaneously (involving a bottleneck), but not when practicing the component tasks in single-task situations. Only the first situation provides participants with sufficient experience to schedule and reschedule the corresponding processes at the bottleneck. (2) As an important precondition for processing two task representations in working memory one should consider the memory load exposed by the two tasks; if it exceeds the necessary memory capacity even after training then one will not find signs for the kind of task coordination improvement predicted by the memory hypothesis. (3) When task representations with a comparable load are processed, then differences in the memory efficiency between participants should be related to the success of improving task coordination by practice. Importantly, the memory hypothesis complements but does not substitute earlier assumptions about practice-related changes in dual-task processing. It shifts the research focus about explaining training-related changes during dual-task processing to the issue of the maintenance and processing of working memory representations. This, in particular, adds to earlier accounts proposing mechanisms such as automatization and optimized resource allocation as main sources for the improvement of dual-task processing (Strobach, 2020; Strobach & Schubert, 2017a).

## Origins of the memory hypothesis in the context of dual-task studies

There are antecedent nonpractice studies that preceded the work on the memory hypothesis, but which supported the foundation of the memory hypothesis for the context of practice studies. De Jong (1995) as well as Hartley and Little (1999) assumed that incomplete instantiation of relevant task information results in impaired dual-task performance. For instance, De Jong (Experiment 1) initiated dual-task trials with cues providing information about the visual or auditory stimulus modality of the first of two overlapping tasks. These cues generated expectancy on the first task (due to a preparatory bias to either process a visual or an auditory stimulus). This expectation could either be valid or invalid, depending on the modality of the upcoming stimulus for the first task: Cues correctly or incorrectly designated the first task, respectively. Under invalid conditions and short intervals between stimulus presentations, the cue rather than the actual stimulus presentation should thus initiate the order in which the two stimuli (and the associated overlapping tasks) are processed. In this case, a central processing limitation (i.e., a bottleneck at response selection) is initially not prepared to process the first presented stimulus and needs to be actively switched. This switch should require an increased processing time for two overlapping tasks under invalid conditions compared with valid conditions. In fact, RTs in the study of De Jong were higher in invalid trials than in valid trials, supporting the assumption of an impact of task instantiation factors on dual-task processing.

Further supporting evidence for the assumption of a fast instantiation of Task 2 processing after the processing of Task 1 has finished, had been provided by dual-task practice studies. In studies of our own group, we compared dual-task performance before and after six sessions of dual-task practice (see Liepelt et al., 2011; Strobach, Frensch, Soutschek, et al., 2012b). The practiced dual task consisted of visual–manual and auditory–verbal choice RT tasks. Note that, in the remaining text, we refer to these input–output modality combinations as the visual and auditory tasks, respectively, and we selected these combinations in such a way that input and output modalities would be different and nonoverlapping between the two component tasks. In the visual task, there were spatially compatible mappings of circle positions (left, central, and right positions) on screen to manual finger responses (index, middle, and ring fingers of the right hand); in the auditory task, different pitches of sinewave tones (low, medium, and high-pitched tones) had a compatible mapping to number words (ONE, TWO, and THREE). The target stimuli were presented simultaneously (i.e., with a stimulus onset asynchrony, SOA, of 0 ms), and prioritization was given equally to both tasks. After six sessions of practice, dual-task performance in the seventh test session improved when compared with the dual-task performance before practice. In more detail, this improvement was mainly demonstrated by reduced dual-task costs in the auditory task, while there was evidence of only a small improvement in the visual task. The auditory task and the visual task are typically performed second and first, respectively, as indicated by longer and shorter RTs (see also Hartley et al., 2011; Hazeltine et al., 2002; Schumacher et al., 2001). The second responses in the auditory task and the earlier first ones in the visual task indicate a second auditory response selection and a first visual response selection (Ruthruff et al., 2003). Thus, faster RTs in the (second) auditory task after practice compared with before practice suggest that particularly auditory response selection might start more efficiently and therefore earlier after dual-task practice.

The assumption of improved task coordination is in line with accounts on dual-task processing that highlight the role of additional executive control processes in dual-task situations even at low practice levels (De Jong, 1995; Hirsch et al., 2018; Kramer et al., 1995; Kübler et al., 2018, 2022a, 2022b; Luria & Meiran, 2003; Sigman & Dehaene, 2006; Strobach et al., 2019; Szameitat et al., 2006). These accounts suggest that bottleneck processing does not result from a passive occupation of the response selection stage by one of the two task processing streams. Instead, the bottleneck processing results from the active and dynamic interaction of basic task processes and of additional executive processes that coordinate both task processing streams at the bottleneck stage. While our previous findings (Liepelt et al., 2011) provided first evidence for the acquisition of improved task coordination skills after dual-task practice, the specific nature of such mechanisms had been subject of further studies focusing on and elaborating the predictions of the memory hypothesis.

## The memory hypothesis

The central assumption of the *memory hypothesis* is that dual-task performance improves with practice because the adopted strategy of coordinating two simultaneous tasks improves the simultaneous preparation of both component tasks that constitute a dual task. This improved preparation is realized by the efficient instantiation of relevant information from these tasks in working memory at the onset of a dual-task trial. We assume that this instantiation primarily involves task-set information about the tasks, including the task-relevant information of stimulus–response (S-R) mapping rules. Importantly, this does not mean that task sets are somehow integrated in working memory (i.e., task integration; Ruthruff et al., 2001, 2006), but efficient instantiation means that tasks are represented separately and at the same time in working memory.

As an example, this efficient instantiation is illustrated in Fig. 1 with an exemplarily shorter visual and a longer auditory task (as realized in Liepelt et al., 2011), in which different processing time durations enable sequential processing of the tasks at the bottleneck. Before practice (Fig. 1B), dual-task processing entails (1) the instantiation of task information for the first (visual) task at the onset of a dual-task trial; (2) the execution of the shorter task with an allocation of the central bottleneck to that task; (3) a subsequent switch of the bottleneck to the longer (auditory) task, which is performed second, including the instantiation of task information for this task (refer to Ruthruff et al., 2001, for an elaborated discussion on some variable time required for participants to switch between tasks); and (4) the execution of this second task. As illustrated in Fig. 1A, there is an efficient (i.e., simultaneous) instantiation of task information for both tasks already at the onset of dual-task trials after dual-task practice. Consequently, the time for a potential switch from the first to the second task is shortened because no instantiation of information for the longer task is required. The shortened switch should primarily lead to advanced dual-task performance on a longer (second) task. It is important to note that these predictions relate to the processing time in the second task but not to the first task; thus, no change in the shorter (first) task performance is predicted, although such changes (e.g., an immediate start of Task 1 processing) are not precluded. These predictions of the memory hypothesis are supported by the empirical findings of Liepelt et al. (2011), and of many other studies (see, e.g., Table 1).

**Table 1:** List of the empirical studies included in the current review, the studies’ main findings, and the related theoretical prediction(s)

Study	Main findings	Related prediction(s)
Liepelt et al. (2011)	Dual-task practice results in improved dual-task performance compared with the results of single-task practice in (1) a practiced dual-task situation, (2) a transfer dual-task situation with a new (unpracticed) visual task, and (3) a transfer dual-task situation with a new (unpracticed) auditory task	Acquisition and transfer of coordination skills due to practice
Maquestiaux et al. (2004)	Dual-task practice results in increased reductions of dual-task costs in younger versus older adults (Experiment 1), while this reduction is similar with less complex tasks (Experiment 2)	Impact of age-related decline in working memory capacity on dual-task optimization
Maquestiaux and Ruthruff (2021)	Dual-task performance is impaired in older adults, even under easy task conditions, because of strategic task differences	Impact of age-related decline in working memory capacity on dual-task optimization (not confirmed)
Ruthruff et al. (2006)	Dual-task practice does not result in improved dual-task performance compared with the results of single-task practice in situation with high complexity and high difficulty	Role of component task complexity and difficulty
Schubert et al. (2017)	Dual-task practice results in improved dual-task performance compared with the results of single-task practice in a transfer dual-task situation with new (unpracticed) visual and auditory tasks	Acquisition and transfer of coordination skills due to practice
Schubert and Strobach (2018)	Dual-task practice results in improved dual-task performance compared with the results of single-task practice in a situation with low complexity and low difficulty (Experiment 1) and in a situation with high complexity and low difficulty (Experiment 2); but there was no such improvement in a situation with low complexity and high difficulty (Experiment 3)	Role of component task complexity and difficulty
Strobach et al. (2014)	Dual-task practice and mixed single-task practice results in improved dual-task performance compared with the results of single-task practice	Acquisition and transfer of coordination skills due to practice
Strobach, Frensch, Soutchek, and Schubert (2012b)	Dual-task practice results in improved dual-task performance compared with the results of single-task practice in (1) a practiced dual-task situation and (2) a transfer dual-task situation with new (unpracticed) visual and auditory task (i.e., there is a trend for improvements in the later situation)	Acquisition and transfer of coordination skills due to practice
Strobach, Frensch, Müller, and Schubert (2012a)	Dual-task practice results in reductions of dual-task costs in younger and older adults	Impact of age-related decline in working memory capacity on dual-task optimization
Strobach, Frensch, Müller, and Schubert (2012b)	Dual-task practice results in reductions of dual-task costs in older adults after extended practice (costs are higher in older vs. younger adults at the end of practice) as well as after extended practice and low difficulty (costs are similar in younger and older adults)	Impact of age-related decline in working memory capacity on dual-task optimization
Strobach, Frensch, Müller, and Schubert (2015a)	Dual-task practice results in improved dual-task performance compared with the results of single-task practice in (1) a practiced dual-task situation, (2) a transfer dual-task situation with a new (unpracticed) visual task, and (3) a transfer dual-task situation with a new (unpracticed) auditory task	Acquisition and transfer of coordination skills due to practice; impact of age-related decline in working memory capacity on dual-task optimization
Strobach, Gerstorf, Maquestiaux, and Schubert (2015b)	Dual-task practice results in reductions of dual-task costs in younger and older adults	Impact of age-related decline in working memory capacity on dual-task optimization
Strobach and Schubert (2017b)	Dual-task practice results in reductions of dual-task costs in younger and older adults (costs are higher in older versus younger adults at the end of practice)	Impact of age-related decline in working memory capacity on dual-task optimization

The memory hypothesis refines other more partial assumptions in this context, such as the allocation and scheduling hypothesis, which was proposed by Strobach (2020), to justify only the observation of an advantage of dual-task training compared with single-task training and the presumed strategy of coordinating two simultaneous tasks resulting from dual-task practice. While this allows for claiming “an advantage in dual-task performance at the end of dual-task practice in comparison with the dual-task performance after single-task practice” (Strobach, 2020, p. 4), it leaves open, by which the exact mechanisms the practice-related improvement in dual-task performance is realized.

The memory hypothesis explains the practice-related improvement by assuming the simultaneous maintenance and timely updating of task representations in working memory. These assumptions add to the assumptions about the processing dynamics at the bottleneck and, thus, provide a more comprehensive picture of what in particular helps to realize the dual-task training advantage compared with single-task training (i.e., Strobach, 2020). As one of these mechanisms the timely improved preparation of task representations at the onset of a dual-task trial could be identified as an important precondition for the realization of the dual-task advantage compared with single-task training (Schubert & Strobach, 2018). In that respect the memory hypothesis provides a broader theoretical frame than the more partial allocation and scheduling hypothesis (Strobach, 2020).

## The memory hypothesis and recent evidence on bottleneck mechanisms

The memory hypothesis focuses on mechanisms causing improved implementation of central bottleneck processing in dual-task situations by active control processes. In that respect, it is important to discuss recent evidence that calls for modifications to the central bottleneck account and to discuss whether alternative accounts would require modifications of the memory hypothesis. For instance, recent evidence suggests the possibility of parallel processing at the level of the central stage (Fischer & Plessow, 2015; Koch et al., 2018; Musslick & Cohen, 2021) and that the motor level is a potential additional (or alternative) source of dual-task interference (Klapp et al., 2019). According to such evidence, one could assume that dual-task performance after practice is not only improved due to an efficient instantiation of relevant task information in working memory at the onset of a dual-task trial (i.e., the memory hypothesis) but due to other mechanisms as well.

As one opportunity, one should consider that a stronger practice-related dual-task improvement for Task 2 than for Task 1 (as is interpreted as evidence for improved task coordination) could reflect stronger parallel processing of Task 2 during Task 1. In our view, such an account is possible to some extent, but it cannot completely explain the full set of findings reported in the current review, and it is not the only mechanism explaining improved dual-task performance after training. In more detail, Maquestiaux and colleagues (Maquestiaux et al., 2008, 2018) demonstrated that parallel processing and, thus, bypassing the central bottleneck are possible under particular conditions after single-task practice in younger adults (i.e., stronger parallel processing of Task 2 during Task 1 processing). However, as outlined below, single-task practice is insufficient to enable practice-related changes at the bottleneck mechanism. Thus, Liepelt et al. (2011) and further studies (see Table 1, Strobach, 2020) demonstrated a clear disadvantage in dual-task performance after single-task practice in comparison to dual-task practice and reported tremendous dual-task costs even after the former type of practice, which suggests that the task situations in our studies have precluded practice-related changes leading to a bottleneck bypass. This indicates that studies investigating bottleneck bypassing seem to focus on practice mechanisms different from those proposed by the current memory hypothesis. Thus, potential evidence for stronger parallel processing of Task 2 during Task 1 processing does not exclude the existence of mechanisms associated with the latter hypothesis.

A further finding supports the conclusion that the presumed practice-related changes of task coordination are distinguishable from the mechanisms on bottleneck bypassing. While the latter phenomena are usually shown for changes within the practiced tasks (for a recent detailed elaboration on these task-specific practice effects, see also Garner & Dux, 2023), the changes associated with the improvement of task coordination seem not necessarily be associated with the specifically practiced tasks, but the skills can be transferrable to tasks with other stimuli and mappings. Liepelt et al. (2011) and other studies (Strobach, Frensch, Soutschek et al., 2012b, Strobach, Frensch et al., 2015a, Schubert et al., 2017) showed a preserved dual-task advantage compared with single-task practice even if the tasks were changed after training with respect to stimuli and response mappings. This indicates that the acquired executive skills are (at least) to some extent transferrable to new task situations and not associated with the specifically practiced stimuli. In sum, both types of practice-related changes are conceivable and exist as distinguishable mechanisms.

The set of findings of Liepelt et al. (2011) is also inconsistent with a further alternative explanation. According to that, improved dual-task performance after practice could result from a change in sharing the limited processing capacity between the component tasks (e.g., Logan & Gordon, 2001; Meyer & Kieras, 1997; Miller et al., 2009; Navon & Miller, 2002; Tombu & Jolicœur, 2003). Dual-task practice might then result in improved Task 2 performance at the end of practice through an increase in parallel processing of Task 1 and Task 2 processes and an increased consumption of the limited processing capacity available for Task 2 processing. However, such a cause for the improvement in Task 2 performance should be accompanied by the simultaneous impairment in Task 1 performance (Tombu & Jolicœur, 2003). When elaborating on the results of Liepelt et al., however, there is no such impairment in Task 1 performance after dual-task practice in comparison with the consequences of single-task practice. So, the study of Liepelt et al. provided evidence that is not consistent with explanations assuming an increased amount of capacity sharing after practice as the core of ongoing practice-related changes.

Finally, the memory hypothesis focuses on mechanisms related to executive control at the central bottleneck mechanism; it does not exclude that interference may occur at other processes during dual-task processing, such as at the motor level (Klapp et al., 2019). Nevertheless, the component tasks applied in the studies supporting the current memory hypothesis usually excluded interference at the motor level by applying motor responses in different motor modalities (e.g., manual and verbal effectors).

## Theoretical aims and empirical investigations on the memory hypothesis

Although previous findings (Hazeltine et al., 2002; Liepelt et al., 2011; Ruthruff et al., 2006; Schumacher et al., 2001) indirectly supported the assumptions of the memory hypothesis, a more comprehensive overview of the work on this hypothesis is still pending, which at the same time can serve as an assessment of the available evidence. Thus, the aim of the current paper is to elaborate on the validity of the memory hypothesis. For this purpose, we review empirical findings on three critical predictions, which can be derived when putting assumptions about working memory together with assumptions about the dynamics of coordination and training-related changes in dual-task processing. These predictions concern (1) the preconditions for the acquisition and transfer of coordination skills due to practice, which should optimally reflect the essentials of task coordination as given by comparing dual-task and single-task practice situations; (2) the role of component task complexity and difficulty in the efficient instantiation of task representations in working memory (if the information load of the memory representations exceeds memory capacity, then this should impair their successful handling in working memory); and (3) the potential impact of a decline in participants’ working memory capacity (operationalized by age-related changes) on the success of practice-related dual-task optimization. Participant cohorts characterized by working memory impairments should expose specific difficulties in preparing task representations during dual-task processing. The theoretical predictions and related empirical tests will be outlined in more detail below.

To review the memory hypothesis systematically, we conducted a literature search in the abstract databases PubMed and PsycINFO in October 2022. For this search, we used the following combination of search terms ((“dual task” OR “dual tasks”) AND (training OR practice)), resulting in 1,583 (PubMed) and 708 (PsycINFO) peer-reviewed, empirical studies in English. A total of 387 studies appeared in both searches. After screening titles, abstracts, and method sections, 13 studies were included in the current review of the memory account (for an overview, see Table 1).

## Preconditions for the acquisition and transfer of coordination skills due to practice

### Theoretical predictions

According to the memory hypothesis, dual-task practice results in an efficient instantiation of task information for both tasks at the onset of dual-task trials. As a result, there is no requirement for a time-consuming instantiation of the second task set after the first task has been processed at the bottleneck stage, which, as a net effect, decreases the assumed time to switch between tasks at the bottleneck. The shortened switch should primarily lead to advanced dual-task performance in the second task (as will be specified below). In contrast, single-task practice, where two component tasks are practiced separately, should not lead to an efficient instantiation of task information for both tasks at the onset of a dual-task trial. This is so because on single-task trials during this practice type, only one task is performed, and thus, the instantiation of a second task set would not be beneficial and cannot be trained. Rather, single-task practice teaches to use up the entire working memory for one task (just because its available), while dual-task practice teaches to build task-set representations in a way that allows to employ the available capacity more efficiently. Therefore, after single-task practice, participants still only activate one task set at the beginning of a dual-task trial (similar to Fig. 1B). This requires an additional instantiation process after the completion of the central processing stage in the first task and before switching to that stage in the second task. Hence, dual-task practice compared with single-task practice should result in larger improvements in dual-task performance, particularly in Task 2. By comparison, extended single-task practice might lead to better preparation of Task 1 processes, but it should leave Task 2 processes rather unchanged due to a lack of experience with that task.

Furthermore, the assumption of the efficient instantiation of task information and the shortened switching operation as an explanation for dual-task optimization can be distinguished from other explanations focusing on the integration of two tasks into a combination of, in an extreme case, a single super task (Hazeltine et al., 2002), as well as task automatization and/or stage shortening within the component tasks (e.g., Ruthruff et al., 2006). While the latter explanations (super tasks, automatization, and stage shortening) propose that improvements are specific to the practiced tasks, the former explanation (the memory hypothesis) suggests that improved task coordination skills for efficient task instantiation could be transferred to dual-task situations with a different component task composition. In detail, skills for efficient instantiation of task information and the improved switching operation are assumed to not be specifically tied to the perceptual information, S-R information, and motor information of the particular tasks presented during practice. Instead, the memory hypothesis predicts that dual-task practice could be generalized to some extent to other stimuli and component task information in structurally similar dual-task situations. Thus, this type of practice should also result in improved performance in new and untrained dual-task situations. Such independence from the specifically practiced tasks is consistent with assumptions of generally improved task coordination skills after dual-task practice (e.g., Anguera et al., 2013; Bherer et al., 2006; Kramer et al., 1995) and after other types of executive control training (Karbach & Strobach, 2022; Karbach et al., 2015; Karbach & Verhaeghen, 2014; Nguyen et al., 2019). Interestingly, such independence of the acquired skills from the processed stimuli and tasks is not predicted by mechanisms associated with accounts such as the bottleneck bypassing account and other accounts proposing rather task-specific practice-related changes.

### Empirical tests

Strobach et al. (2014) offered a design to test the memory hypothesis by comparing the effects of single-task practice and dual-task practice on dual-task performance. According to the memory hypothesis, dual-task practice (compared with single-task practice) should result in an efficient task set instantiation for both component tasks at the beginning of a dual-task trial and, thus, a faster switch from Task 1 to Task 2 at the bottleneck stage. As a result, RTs for Task 2 should be reduced after dual-task practice compared with single-task practice. However, efficient task set instantiation for both component tasks at the beginning of a dual-task trial could also result from other types of practice in which both component tasks have to be prepared for all trials.

As a result, in a pretest session of this study, participants performed a dual task consisting of a visual Task 1 and an auditory Task 2. For both tasks, participants had to maintain three S-R mappings active in working memory. In addition to dual-task performance, we also measured single-task performance for each component task. After this pretest session, participants were assigned to three different practice groups. During five practice sessions, all three groups received the same number of Task 1 trials and Task 2 trials (465 trials per task per session); however, the groups received these tasks in different contexts. In the *single-task group*, participants practiced both component tasks in isolation (i.e., both tasks were presented in separate blocks). In the *dual-task group*, participants performed five sessions of dual-task practice (i.e., simultaneous practice of both tasks). Importantly, in the *mixed single-task* group, participants also practiced only single tasks. However, the tasks were presented intermixed and randomly within the same blocks, so participants had to prepare for both tasks on every trial, despite performing only one task in each trial. In the final session, all groups performed single- and dual-task blocks.

The results showed that RTs for the second auditory task were reduced in the dual-task practice group and the mixed single-task practice group compared with the single-task practice group in the final session. A comparison of the specific magnitudes of the costs in the auditory task RTs after dual-task practice/after mixed single-task practice with the costs after single-task practice shows that the dual-task group/mixed single-task group produced only 21% and 29% of the costs in the single-task group, respectively. Performance for the auditory task in single-task blocks and performance for the visual task in single- and dual-task blocks did not differ between groups. This observation supports the memory hypothesis, according to which only after practice where both tasks are relevant within the same blocks, participants are able to efficiently instantiate task information for both component tasks. However, performance on the first visual task did not differ after the different types of practice, which provides further evidence that is inconsistent with predictions of capacity sharing models on dual-task practice (the latter models would predict an impairment after dual-task practice in the first task performance, as described in the introduction).

In Liepelt et al. (2011) as well as Strobach, Frensch, Soutschek, et al. (2012d), we tested whether the improvements in dual-task coordination skills and the efficient instantiation of two tasks are transferrable to other task situations; according to the memory hypothesis, dual-task practice should also result in improved performance in new and untrained dual-task situations. Therefore, we tested for the transfer effects of dual-task practice. For this purpose, we compared dual-task performance in a *single-task group* and a *dual-task group* before and after practice. The dual task consisted of a Visual Task 1 and an Auditory Task 2, with three S-R mappings for each task. While the dual-task group practiced the dual task over the course of several sessions, the single-task group practiced the component tasks in isolation. In addition to the trained dual task, in the posttest we also applied a transfer dual-task situation in both groups. In this transfer dual task, we applied new component tasks that differed with respect to the presented stimuli as well as the applied S-R mapping compatibility in the visual task, in the auditory task, or in both tasks.

As a result of applying a new visual task (with an old and practiced auditory task) or a new auditory task (with an old and practiced visual task) after the end of practice during transfer (Liepelt et al., 2011), we found strongly improved dual-task performance in auditory Task 2 after practice only in the dual-task group but not in the single-task group. In the transfer situation, a comparison of the specific magnitudes of the costs in the auditory task RTs of the dual-task groups and of the single-task groups shows that the dual-task groups produced only 69% and 35% of the costs in the single-task groups when the visual task or the auditory task was new, respectively. Performance on the visual task did not differ between groups. This finding suggests that dual-task practice (but not single-task practice) results in a faster switch from Task 1 to Task 2. More importantly, we found evidence for the fact that improved task instantiation and associated coordination skills can also be transferred to similar dual tasks with a different task composition. These findings support the memory hypothesis, which predicts that dual-task practice should also result in improved performance in new and untrained dual-task situations.

**Fig. 2:**
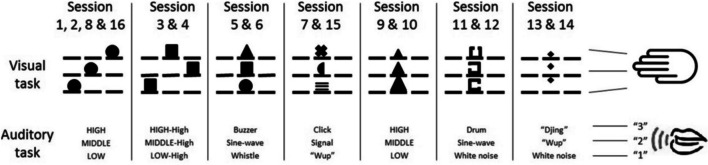
Illustration of the stimulus and the stimulus–response mapping characteristics of the visual and auditory tasks in the dual-task and single-task groups across Sessions 1–16 in the study of Schubert et al. (2017)

However, when applying a new visual task and a new auditory task during transfer (Strobach, Frensch, Soutschek et al., 2012d), improved dual-task performance after dual-task practice was in a less clear manner; it led merely to reduced error rates in the Auditory Task 2 in the dual-task group (but not in the single-task group) after practice. Because of the less clear results when applying two new tasks simultaneously during transfer (Strobach, Frensch, Soutschek, et al., 2012d) and, in order to test the limits of the transfer of improved task instantiation, Schubert et al. (2017) compared dual-task performance in a Visual Task 1 and an Auditory Task 2 before and after 16 sessions of single-task practice and dual-task practice. In that study, participants did not perform the same visual and auditory tasks throughout practice, but they received practice with increased variability between task situations across different training sessions. That means stimulus and S-R mapping conditions in the two component tasks changed repeatedly across the practice sessions (Fig. 2), which prevents automatized S-R associations from being transferred from practice to transfer. The repeated changes also increased the variability of practice and might thus enforce the transferability of improved task instantiation skills (Schmidt & Bjork, 1992).

Dual-task performance was found to improve after practice in the dual-task group in contrast to the single-task group. In more detail, similar to the previous studies, improved dual-task performance was indicated by lower dual-task RT costs in the longer Auditory Task 2 (in detail, the dual-task group produced only 36% of the costs of the single-task group), which is consistent with the assumption of a faster switching process from Task 1 to Task 2 at the bottleneck stage after practice. These findings are consistent with the prediction of the memory hypothesis, assuming that improved task instantiation can be transferred to other dual-task situations independently of the specific stimulus and S-R mapping conditions of the practiced component tasks. In total, the findings of this section illustrate the preconditions for the acquisition and transfer of coordination skills through practice. In contrast, these findings are inconsistent with accounts such as the bottleneck bypassing account and accounts assuming the combination of single super tasks, which assume that practice-related changes due to repeated dual-task performance occur primarily as a result of task-specific changes in the component tasks.

An issue needs to be discussed, which relates to the assumption of the improved switching operation between tasks at the bottleneck. On one side, the memory hypothesis could theoretically be restricted to the assumption that dual-task practice leads simply to the efficient, simultaneous instantiation of two task sets in working memory, thus allowing for improved preparation of both tasks at the onset of a dual-task trial. In other words, this account would leave out the idea of a practice-related, improved switching operation. However, this view might not reflect the complexity of practice-related changes during extended training. Thus, studies with a related paradigm, in which participants switch repeatedly between two component tasks in several sessions (i.e., task switching), have often shown that the time to switch between tasks per se can be shortened after appropriate training (Kray & Lindenberger, 2000; Strobach, Liepelt et al., 2012e). Several studies even reported evidence that switching processes can be improved on a task-nonspecific level, which suggests the possibility of a generalized practice-related improvement of these processes. This would be consistent with the assumption that dual-task practice leads to an improvement of switching between the task representations on the process level and that this improvement accompanies or even enables the changes that lead to the efficient instantiation of the two component tasks in working memory. Since the findings on transferable, practice-related changes in Task 2 processing (Liepelt et al., 2011; Schubert et al., 2017; Strobach, Frensch, Soutschek, et al., 2012d, and others) are consistent with both possibilities, the memory hypothesis covers the possibility of improved executive control during switching between task representations. In fact, the assumption that dual-task practice leads to a task-nonspecific improvement of the switching operation allows for the prediction that appropriate training designs with dual-task situations can lead to practice effects generalizable to task-switching situations and vice versa (Strobach, Frensch, & Schubert, 2012c).

## Role of component task complexity and difficulty

### Theoretical predictions

A key assumption of the memory hypothesis is that, after dual-task practice, participants are enabled to instantiate both component task sets in working memory at the beginning of a dual-task trial. However, given the limited capacity of working memory (e.g., Cowan et al., 2005; Oberauer, 2009), instantiating two task sets concurrently may only be possible in situations that do not exceed the processing capacities of this memory component. Different factors relating to the complexity of the component tasks, such as S-R mapping compatibility (e.g., Ruthruff et al., 2006) or the number of S-R mappings that must be kept active in working memory (i.e., difficulty; e.g., Stelzel et al., 2008) can influence the processing demands on working memory in dual-task situations. Thus, introducing more complex or difficult tasks (i.e., tasks with incompatible or a higher number of S-R mappings, respectively) can serve as a critical test for the memory hypothesis. In more detail, according to this hypothesis, performing more complex/difficult component tasks compared with relatively simple/easy component tasks may exceed working memory capacity. As a result, participants are not able to efficiently instantiate the task sets of the component tasks at the beginning of a dual-task trial. Importantly, this would lead to a reduction in practice effects compared with dual-task situations with relatively simple/ easy component tasks.

### Empirical tests

In Schubert and Strobach (2018), we investigated the role of task complexity and difficulty in the effect of practice on dual-task performance. According to the memory hypothesis, the effects of dual-task practice depend on available working memory resources. As a result, if the demands of the component tasks exceed working memory capacity, participants cannot instantiate the task sets of the component tasks concurrently. This is particularly the case when assuming that participants represent the entire task but not only parts in working memory, or they do not represent them at all (however, see the Summary section). Thus, there should be no additional practice effects of dual-task practice over single-task practice in dual-task situations with demanding and more complex/difficult component tasks.

In Experiment 1 of Schubert and Strobach (2018), we applied a dual task consisting of an auditory task with a set of two compatible S-R mappings and a visual task with a set of four compatible S-R mappings. A dual-task group practiced this dual-task situation over the course of nine sessions. A single-task group practiced the same tasks in isolation under a single-task condition. In the test session after practice, dual-task RT costs on Task 2 were 59% lower after dual-task practice than after single-task practice, which indicates the efficient instantiation of relevant task information at the beginning of a dual-task trial.

In Experiment 2, we tested the boundary conditions of S-R mapping compatibility and thus task complexity in the component tasks for efficient task set instantiation since task compatibility might expose a critical burden for dual-task processing under conditions of limited capacity of the working memory. Therefore, we changed the compatibility level of Experiment 1 (i.e., two component tasks with compatible S-R mappings) to a practice situation with one compatible Task 1 and one incompatible Task 2 in Experiment 2. Importantly, despite this change in our practice regimen, we found that dual-task (in contrast to single-task) practice resulted in lower RT costs for the second task, replicating the findings of Experiment 1 (in detail, the dual-task group produced only 40% of the costs of the single-task group). Hence, we demonstrated that the efficient instantiation of both task sets at the beginning of a dual-task trial is possible even under conditions of increased task complexity and their increased demands on participants’ processing capabilities, which are exposed by the need to process a mix of compatible and incompatible S-R mappings in the component tasks.

In Experiment 3, we aimed to test whether an increased number of S-R mappings and thus increased difficulty represent a limiting factor preventing efficient task instantiation. The number of S-R mappings to be held online may be restricted given the limited capacity of the working memory used during task processing (e.g., Oberauer, 2009). Thus, increasing the number of S-R mappings might exceed the necessary working memory capacity and prevent the efficient instantiation of the task information for both tasks. To test this assumption, we increased the number of S-R mappings from two and four for the auditory and visual tasks, respectively (as in Experiments 1 and 2), to two and eight S-R mappings for the auditory and visual tasks, respectively. Importantly, the results of Experiment 3 did not provide any evidence for a difference between the practice effects in the dual-task and single-task groups. Thus, under conditions of an increased number of S-R mappings (compared with Experiments 1 and 2), we could not find evidence for efficient task instantiation of task 2 after dual-task practice. This is consistent with the assumption that the task difficulty in Experiment 3 has exceeded the available capacity of the working memory in a way that prevents the efficient instantiation of the component tasks during dual-task processing.

These findings of Schubert and Strobach (2018) are consistent with other dual-task practice studies. For instance, the efficient instantiation of task information was impaired when highly complex and highly difficult tasks were presented under dual-task practice conditions (Ruthruff et al., 2006). Specifically, there was no advantage of dual-task practice in contrast to single-task practice regarding dual-task performance with component tasks that included a high number of S-R mapping rules. These tasks included four and eight mapping rules in a first auditory and a secondary visual task, respectively. Furthermore, the rule sets created a mix of compatible and incompatible mappings. This high number and mix of rules may have resulted in a working memory overload and therefore may have hampered the efficient instantiation of task information that is needed to induce practice-related benefits in terms of dual-task performance. In sum, these findings of Ruthruff et al. validate the role of component task complexity and difficulty in the context of the memory hypothesis.

Based on the findings of Schubert and Strobach (2018) and Ruthruff et al. (2006), it is tempting to specify the limits of the working memory load, which can be considered a break point for the efficient instantiation of the task sets after practice. If one presumes the number of S-R mappings as separable units independently of the tasks, then the findings of Schubert and Strobach (2018, Experiments 2 and 3) allow for a specification of that breakpoint between 6 and 10 S-R mappings. However, we are aware that the maintenance of S-R mappings in working memory might be tremendously affected by the possibility of efficient memory organization of the mappings by chunking and other factors such as task rule systematization (Duncan, 1979). Even the way of learning rule might play a role (Dreisbach & Wenke, 2011), and, finally, one has to take into account individual differences in the working memory capacity between participants (e.g., Broeker et al., 2022) when aiming to specify the break point.

## Impact of age-related decline in working memory capacity on dual-task optimization

### Theoretical predictions

An additional aim of this review is to summarize findings that test our model in individuals with deficits in executive functioning, including decreased working memory capacity. For this purpose, we applied our dual-task practice regimen to the elderly (Krampe et al., 2005; Lindenberger et al., 1992). Prior research has shown that older adults show increased dual-task costs compared with younger adults (e.g., Glass et al., 2000; Hein & Schubert, 2004; Verhaeghen et al., 2003). According to some accounts, these increased dual-task costs can be explained by a decreased ability to maintain task information active in working memory and, thus, a slower switch from the first to the second task at the bottleneck stage (e.g., Hartley & Little, 1999; Maquestiaux et al., 2004). Concerning potential practice effects, the memory hypothesis predicts that a lower working memory capacity should result in decreased optimization of dual-task performance due to practice. This is so because the instantiation of task information at the beginning of a dual-task trial depends on available working memory resources. In particular, if the working memory capacity is not sufficient for maintaining two task representations simultaneously, then an efficient task instantiation for both tasks cannot take place at the beginning of a dual-task trial. Given their lower working memory capacity, older adults should not only show decreased performance before practice compared with younger adults. They should also capitalize to a lesser degree on dual-task practice because of the lower opportunity to maintain two task representations simultaneously after practice compared with younger adults. While this assumption presumes that efficient task representations will be activated completely as a whole but not partially, it predicts that, when comparing older and younger adults, the former (versus the latter) should show reduced practice gains after dual-task practice.

In addition, research on age-dependent decline has shown that older adults can show similar performance levels compared with younger adults when demands on the tasks are reduced (Maquestiaux et al., 2004; Reuter-Lorenz & Cappell, 2008). This observation has often been explained by the fact that low-demand tasks (e.g., easy and simple tasks) allow for a compensatory adaptation of task processing. For high-demand tasks (e.g., complex and difficult tasks), on the other hand, age-related deficits cannot be compensated for, resulting in performance differences between different age groups after practice. In analogy, it is also possible that, when memory demands of the component tasks are low, older adults can capitalize on dual-task practice as much as younger adults do. As a result, older adults should show similar practice gains compared with younger adults after practice of a dual-task situation with reduced demands in the component tasks if dual-task impairments do not result from other sources than a decreased working memory capacity.

### Empirical tests

In Strobach et al. (2012a), we investigated whether and how changed task scheduling and/or the acquisition of task coordination skills in different age groups modulate the effects of dual-task practice. For this purpose, we compared the dual-task performance of younger adults (i.e., 19–29 years) and older adults (i.e., 57–68 years) before and after dual-task practice. Both groups performed a dual task consisting of a Visual Task 1 and an Auditory Task 2. Practice was applied over the course of eight sessions. After practice, younger adults mainly showed faster RTs compared with before practice in the longer Auditory Task 2 but only a small improvement in the Visual Task 1, indicating the efficient instantiation of component task information at the beginning of each trial and replicating earlier findings (e.g., Liepelt et al., 2011). Importantly, we found a strikingly similar pattern for the older participants (i.e., reduced dual-task RTs mainly in the auditory, but not in the visual, task after practice; see also Strobach, Gerstorf et al., 2015b; Strobach & Schubert, 2017b). This finding suggests that the mechanisms underlying improved dual-task performance with practice might be similar in older and younger adults. Importantly, however, at the end of practice, dual-task costs were still consistently higher in older adults compared with younger adults (in detail, the younger group produced only 14% of the costs of the older group). Thus, in line with the memory hypothesis, it seems that older adults do not achieve the same dual-task performance level as younger adults at the end of practice, presumably due to decreased working memory capacity.

In Strobach, Frensch et al. (2015a), we tested the potential practice and transfer effects of dual-task practice on task coordination skills for improved task instantiation in older adults. For this purpose, in Experiment 1, we applied a dual task consisting of a shorter Visual Task 1 and a longer Auditory Task 2. A dual-task group practiced this dual task over the course of eight practice sessions. A single-task group only practiced the component tasks in isolation. After practice, the dual-task group, compared with the single-task group, showed a clear reduction in dual-task RT costs in the auditory (but not in the visual) task (in detail, the dual-task group produced only 41% of the costs of the single-task group in the auditory task after practice), indicating the efficient instantiation of component task information at the beginning of each trial even in older adults.

In Experiment 2, we tested for potential skill transfer effects in older adults. For this purpose, both the dual-task and the single-task groups performed eight practice sessions. In Session 9, we changed the compatible S-R mapping of the auditory task to an incompatible mapping under the first transfer condition, and in the second transfer condition, we applied a new stimulus and mapping set for the visual task. Importantly, under both transfer conditions, we found clearly reduced dual-task RT costs for the Auditory Task 2 in the dual-task group (but not the single-task group) despite the significant changes in the component tasks (in detail, the dual-task group produced only 80% and 60% of the costs of the single-task group when the visual task or the auditory task was changed, respectively). Thus, the findings of this study demonstrate not only the acquisition of task coordination skills during practice but also their transferability to new dual-task situations in older adults.

In Strobach et al. (2012b), we investigated whether or not older adults’ dual-task performance can be improved due to extended practice. Furthermore, we investigated the limitations of practice-related dual-task improvements in older adults. In particular, we tested for the achievement of a practice state, which we called nearly perfect time sharing (optimal dual-task performance as achieved by some younger adults; Schumacher et al., 2001; Strobach & Schubert, 2017b), which is characterized by nearly no dual-task costs in the dual-task conditions compared with the single-task conditions. We tested the limits in older adults under conditions of (a) a high amount of dual-task practice (22 sessions compared with eight sessions in younger adults) and (b) practice with less difficult (i.e., easier) component tasks in dual-task situations (two S-R mappings compared with three S-R mappings per task in younger adults). The data showed that a high amount of dual-task practice did not allow older adults to improve dual-task performance up to the optimal level that was achieved by younger adults with a far lower amount of practice; in detail, the younger group produced only 35% of the costs of the older group (compared with 14% after eight sessions). However, practice with easier component tasks in dual-task situations exclusively in older adults provided a similar level of optimal dual-task performance in both age groups. Therefore, through applying a testing the limits approach, we demonstrated that older adults improved dual-task performance to the same level as younger adults at the end of practice under very specific conditions. Under these very specific conditions, there might be an application of efficient task instantiation in older adults in the same way as in younger adults.

Maquestiaux et al. (2004) reported findings in older adults that were consistent with these assumptions. In their Experiment 1, dual-task costs in a second task were reduced by only 32% with the practice of two simultaneous tasks; in this case, the tasks were highly complex and thus had a high load on working memory capacity in older adults. In younger adults, there was a substantial practice-related reduction of these costs by 69%. These differential effects across age groups are consistent with the assumption of Maquestiaux and colleagues that, with nonimpaired working memory functioning, participants were able to learn to efficiently initiate a second task. Older adults with impaired working-memory function were not able to learn this instantiation of a complex second task unless its complexity, and therefore the associated working memory load, was reduced (Maquestiaux et al., 2004, Experiment 2). In fact, there might be additional factors beyond pure differences in working memory capacity, which can additionally affect the comparison of practice- and age-related effects on dual-task performance and cause findings discrepant to the assumptions above. For example, in a recent study, Maquestiaux and Ruthruff (2021) reported increased dual-task costs in older adults in situations in which Task 2 was incredibly easy. The authors explained these findings with changed task strategies in older participants in the easy task condition, with a bias toward a too cautious handling of the serial processing mechanism at bottleneck processing in order to compensate for perceived age-related cognitive slowing. While this explanation focuses on strategic differences in dual-task processing, the current memory hypothesis refers to lower working memory capacity as the cause of different practice effects depending on age.

To sum up, the fact that we observed that older adults improved dual-task performance to the same level as younger adults at the end of practice under very specific conditions only is consistent with the predictions of the memory hypothesis for practice-related dual-task optimization. The memory hypothesis suggests that under conditions of decreased memory load in a dual-task situation, differences in working memory capacity should not be decisive for the acquisition of practice-related dual-task optimization (if other factors do not confound). From a broader perspective, the current findings are important for research on age-related plasticity of the cognitive system because they suggest that an age-related decline of cognitive processing capabilities can be compensated to some degree (but not fully) with appropriate practice and under conditions of appropriate task complexity.

## Summary

The present article reviews empirical findings on the memory hypothesis, a specific mechanism explaining improved dual-task performance after practice. As a central assumption of this hypothesis, we assume that dual-task performance improves with dual-task practice because of the efficient instantiation of relevant task information in working memory at the onset of a dual-task trial. We assume that this instantiation primarily involves task-set information, including task-relevant information about S-R mapping rules. For this purpose, we reviewed the study findings on three critical predictions. These predictions concerned (1) the preconditions for the acquisition and transfer of coordination skills due to practice; (2) the role of component task complexity and difficulty; and (3) the impact of age-related decline in working memory capacity on dual-task optimization. The reported findings show evidence supporting the assumption that practice-related changes during repeated dual-task performance can lead to changes in the memory organization of component task processing during dual-task processing. We found evidence for all predicted changes in the memory organization of task processing across different types of situations and for different age groups. In future studies, it would be additionally interesting how different practice-related mechanisms such as the bypassing mechanism (e.g., Lyphout-Spitz et al., 2022; Maquestiaux et al., 2008, 2010, 2013, 2018; Ruthruff et al., 2006) and the mechanisms of the memory hypothesis (e.g., Liepelt et al., 2011; Schubert et al., 2017; Schubert & Strobach, 2018; Strobach, Frensch, Soutschek et al., 2012d, Strobach et al., 2014, Strobach, Frensch et al., 2015a) could be investigated in a combined manner. This would allow us to disentangle their individual contributions to and their boundaries for the occurrence of practice-related dual-task improvements; this would also allow for a comprehensive investigation of practice mechanisms to explain improved dual-task performance and for the generation of a comprehensive change model in the field. For instance, it would be relevant to apply component tasks that have proven a potential for bottleneck bypassing after single-task practice (Maquestiaux et al., 2008, 2018) in a dual-task practice regime and compare the effects in this regime with the effects in a single-task practice regime.

Future studies should also elaborate on the exact nature of task information and, thus, task representations that are efficiently instantiated at the beginning of a dual-task trial. For instance, it is open whether, after dual-task practice, the entire task sets are represented as a whole in working memory or whether their partial processing and maintenance in working memory is possible, and if so, under what conditions. Similarly, recent studies with nonpracticed dual-task situations have provided evidence for the existence of abstract task-order sets, which guide processing order at the bottleneck in dual-task situations with randomly changing order of the component tasks (Kübler et al., 2022b). These findings indicate that the task-order set is maintained together with the task sets of the component current in working memory during task processing. In the context of the memory hypothesis, it would be intriguing to assess whether extended training with dual tasks with changing processing orders of component tasks would allow for an advanced preparation of two processing orders at the same time, including task-order sets and task sets. This should be indicated by improved dual-task performance in random-order dual task situations compared with training with single-task situations and training with a regular dual-task situation involving a bottleneck but with a fixed order of the tasks.

An important further vein for future research might also consider the issue of the mutual relation between the maintenance of task representations in working memory and their simultaneous execution in dual-task situations and its formalization with appropriate modeling approaches. While formalizing proper maintenance in working memory seems to require assumptions about appropriate memory chunking mechanisms for task representation (Oberauer, 2009; Oberauer et al., 2013), the transition of a task representation into executable process information needs additional assumptions about the timing of cognitive operations and their adjustment during ongoing task processing (Meyer & Kieras, 1997; Musslick & Cohen, 2021; Sigman & Dehaene, 2008). The current findings are consistent with the assumption that extended practice leads to changes in both (i.e., the memory maintenance of the task representation as well as the timing of operations during task execution), which should be considered and integrated by future studies. From a broader perspective, the assumed practice-related changes of the mechanisms as proposed by the memory hypothesis complement other approaches capitalizing on automatization, stage shortening, and/or task integration (Ruthruff et al., 2003, 2006; but see Schneider & Shiffrin, 1977) as important mechanisms for ongoing practice-related changes resulting from dual-task practice.

Finally, it has to be noted that so far, the memory hypothesis had been investigated with dual-task situations consisting of relatively easy component tasks and involving bottleneck processing (Table 1). Therefore, it is unknown to what extent the predictions can be generalized to other dual-task situations consisting of more complex component tasks with higher memory requirements, with changed task compositions, or requiring different types of interference control. This could be specified in future studies.

## Data Availability

Not applicable.
